# Systems genomics in age-related macular degeneration

**DOI:** 10.1016/j.exer.2022.109248

**Published:** 2022-09-13

**Authors:** Anneke I. den Hollander, Robert F. Mullins, Luz D. Orozco, Andrew P. Voigt, Hsu-Hsin Chen, Tobias Strunz, Felix Grassmann, Jonathan L. Haines, Jonas J.W. Kuiper, Santa J. Tumminia, Rando Allikmets, Gregory S. Hageman, Dwight Stambolian, Caroline C.W. Klaver, Jef D. Boeke, Hao Chen, Lee Honigberg, Suresh Katti, Kelly A. Frazer, Bernhard H.F. Weber, Michael B. Gorin

**Affiliations:** a Department of Ophthalmology, Radboud University Medical Center, Nijmegen, the Netherlands; b AbbVie, Genomics Research Center, Cambridge, MA, USA; c The University of Iowa Institute for Vision Research, Iowa City, IA, USA; d Department of Ophthalmology and Visual Sciences, Carver College of Medicine, The University of Iowa, Iowa City, IA, USA; e Genentech, South San Francisco, CA, USA; f Institute of Human Genetics, University of Regensburg, Regensburg, Germany; g Health and Medical University, Potsdam, Germany; h Department of Population and Quantitative Health Sciences, Case Western Reserve University, Cleveland, OH, USA; i Cleveland Institute for Computational Biology, Case Western Reserve University, Cleveland, OH, USA; j Department of Ophthalmology, University Medical Center Utrecht, Utrecht, the Netherlands; k Center of Translational Immunology, University Medical Center Utrecht, Utrecht, the Netherlands; l National Eye Institute, Bethesda, MD, USA; m Department of Ophthalmology, Columbia University, NY, USA; n Department of Pathology and Cell Biology, Columbia University, NY, USA; o Sharon Eccles Steele Center for Translational Medicine, John A. Moran Eye Center, Department of Ophthalmology & Visual Sciences, University of Utah, Salt Lake City, UT, USA; p Departments of Ophthalmology and Human Genetics, University of Pennsylvania, Perelman School of Medicine, Philadelphia, PA, USA; q Departments of Ophthalmology and Epidemiology, Erasmus Medical Center, Rotterdam, the Netherlands; r Institute of Molecular and Clinical Ophthalmology, Basel, Switzerland; s Institute for Systems Genetics, NYU Langone Health, NY, USA; t Department of Biochemistry and Molecular Pharmacology, NYU Langone Health, NY, USA; u Department of Biomedical Engineering, NYU Tandon School of Engineering, Brooklyn, NY, USA; v Gemini Therapeutics, Cambridge, MA, USA; w Department of Pediatrics, University of California, San Diego, La Jolla, USA; x Institute for Genomic Medicine, University of California, San Diego, La Jolla, USA; y Institute of Clinical Human Genetics, University Hospital Regensburg, Regensburg, Germany; z Departments of Ophthalmology and Human Genetics, University of California, Los Angeles, CA, USA

**Keywords:** Age-related macular degeneration, Omics, Systems genomics, Single cell sequencing, Expression quantitative trait locus, Complement system, iPSc-RPE, Induced pluripotent stem cells, Clinical trial, Polygenic risk scores

## Abstract

Genomic studies in age-related macular degeneration (AMD) have identified genetic variants that account for the majority of AMD risk. An important next step is to understand the functional consequences and downstream effects of the identified AMD-associated genetic variants. Instrumental for this next step are ‘omics’ technologies, which enable high-throughput characterization and quantification of biological molecules, and subsequent integration of genomics with these omics datasets, a field referred to as systems genomics.

Single cell sequencing studies of the retina and choroid demonstrated that the majority of candidate AMD genes identified through genomic studies are expressed in non-neuronal cells, such as the retinal pigment epithelium (RPE), glia, myeloid and choroidal cells, highlighting that many different retinal and choroidal cell types contribute to the pathogenesis of AMD. Expression quantitative trait locus (eQTL) studies in retinal tissue have identified putative causal genes by demonstrating a genetic overlap between gene regulation and AMD risk. Linking genetic data to complement measurements in the systemic circulation has aided in understanding the effect of AMD-associated genetic variants in the complement system, and supports that protein QTL (pQTL) studies in plasma or serum samples may aid in understanding the effect of genetic variants and pinpointing causal genes in AMD. A recent epigenomic study fine-mapped AMD causal variants by determing regulatory regions in RPE cells differentiated from induced pluripotent stem cells (iPSC-RPE). Another approach that is being employed to pinpoint causal AMD genes is to produce synthetic DNA assemblons representing risk and protective haplotypes, which are then delivered to cellular or animal model systems.

Pinpointing causal genes and understanding disease mechanisms is crucial for the next step towards clinical translation. Clinical trials targeting proteins encoded by the AMD-associated genomic loci *C3*, *CFB*, *CFI*, *CFH*, and ARMS2*/HTRA1* are currently ongoing, and a phase III clinical trial for C3 inhibition recently showed a modest reduction of lesion growth in geographic atrophy. The EYERISK consortium recently developed a genetic test for AMD that allows genotyping of common and rare variants in AMD-associated genes. Polygenic risk scores (PRS) were applied to quantify AMD genetic risk, and may aid in predicting AMD progression.

In conclusion, genomic studies represent a turning point in our exploration of AMD. The results of those studies now serve as a driving force for several clinical trials. Expanding to omics and systems genomics will further decipher function and causality from the associations that have been reported, and will enable the development of therapies that will lessen the burden of AMD.

## 1. Introduction

Age-related macular degeneration (AMD) is a multifactorial disease, caused by a combination of genetic and environmental risk factors. The strongest environmental risk factors are age and lifestyle factors, such as smoking, obesity, and diet (Lim et al., 2012). AMD has a strong genetic component, with an estimated heritability of 48–71% (Seddon et al., 2005). While the initial identification of genetic loci for AMD was by linkage analysis of sibpairs and extended families and by candidate gene association testing, the most extensive genetic dissection of AMD has been achieved through genome-wide association studies (GWAS) of single nucleotide polymorphisms (SNPs). One of the largest GWAS for AMD to date was performed in 2016, which analyzed DNA samples of 16,144 patients and 17,832 controls (Fritsche et al., 2016). This study identified 52 independent AMD-associated SNPs at 34 distinct genomic loci. Genes at these genomic loci cluster in three main pathways: the complement cascade of the innate immune system, HDL transport, and extracellular matrix organization and assembly. These pathways are important in the pathogenesis of AMD, however, our knowledge about the genetics underlying the causation and progression of the disease is still in its early stage (Yan et al., 2018; Grassmann et al., 2019; Seddon et al., 2020).

An important next step is to understand the functional consequences and downstream effects of AMD-associated genetic variants. For this purpose, other biological molecules can be analyzed, such as DNA modifications (DNA methylation, histone modifications), protein-encoding and regulatory RNA transcripts, proteins and metabolites (substrates, metabolic intermediates including carbohydrates, peptides, lipids and combinations of these constituents). To do this in a systematic and comprehensive way, ‘omics’ technologies can be employed. Omics refers to the recently developed but widely used high-throughput characterization and quantification of biological molecules. These analyses are usually agnostic to the underlying biological mechanism and therefore effective in identifying new candidate genes, proteins or metabolites associated with disease mediation. Multifactorial diseases like AMD are difficult to assess using classical low-throughput methods, since this often requires assumptions about possible candidate genes, and such focused and detailed functional analyses are labor- and time-consuming and due to intrinsic dependency on model systems (e.g. *in vitro* models, animal models) is often conceptually limited. Another advantage of omics studies is that its high-throughput design allows large case-controlled cohorts that overcome sample heterogeneity and generates sufficient power to map the universe of disease driving factors that hallmark multifactorial diseases. These types of datasets can subsequently be employed to guide targeted or mechanistic experiments to further investigate the causal role of certain genes or proteins of interest in the disease.

Omics can be performed on different biological molecules; most commonly used are DNA (genomics), RNA (transcriptomics), proteins (proteomics), metabolites (metabolomics) and lipids (lipidomics). AMD is well-studied on a genomic level using candidate gene assessment and GWAS (Fritsche et al., 2016). Next to this, several other omics have been used in AMD research, including epigenomics (large-scale analysis of epigenetic modifications on DNA or histones), transcriptomics, miRNA profiling, proteomics, metabolomics, and lipidomics (Lauwen et al., 2017). Collectively, these approaches point towards immune-mediated mechanisms underlying the etiology of AMD. This is in agreement with the genomic studies, since the complement system is part of the innate immunity and its activation stimulates systemic and local inflammation (de Jong et al., 2021). Furthermore, oxidative stress-related factors have been regularly identified to be upregulated and oxidative protein modifications were found in proteomic analysis of drusen (Lauwen et al., 2017). These findings suggest that inflammation and oxidative stress are important contributors to AMD pathogenesis, as they are to other age-related diseases.

Building on those results, systems genomics aims to integrate genomics with multiple types of omics data to study the downstream effects of genetic variation. Ultimately, this will lead to a better understanding of the processes that are involved in the disease, will improve the predictive accuracy of AMD susceptibility and disease progression, and will identify potential therapeutic targets. The Systems Genomics task group of the Ryan Initiative for Macular Research (RIMR) aimed to provide direction on how omics data can be used to better understand the pathogenesis of AMD. Between February and April 2021, the task group convened in three virtual sessions. During these sessions, discussions focused on different approaches that have recently been used to decipher the effect of AMD-associated genetic variants on the disease mechanisms, and how genetic contributions of AMD can be incorporated into patient care and clinical trials. This review provides a summary of the discussions held during the RIMR Systems Genomics task group meetings.

## 2. Opportunities and challenges to learn from genome-wide association studies

**Fig 1.**
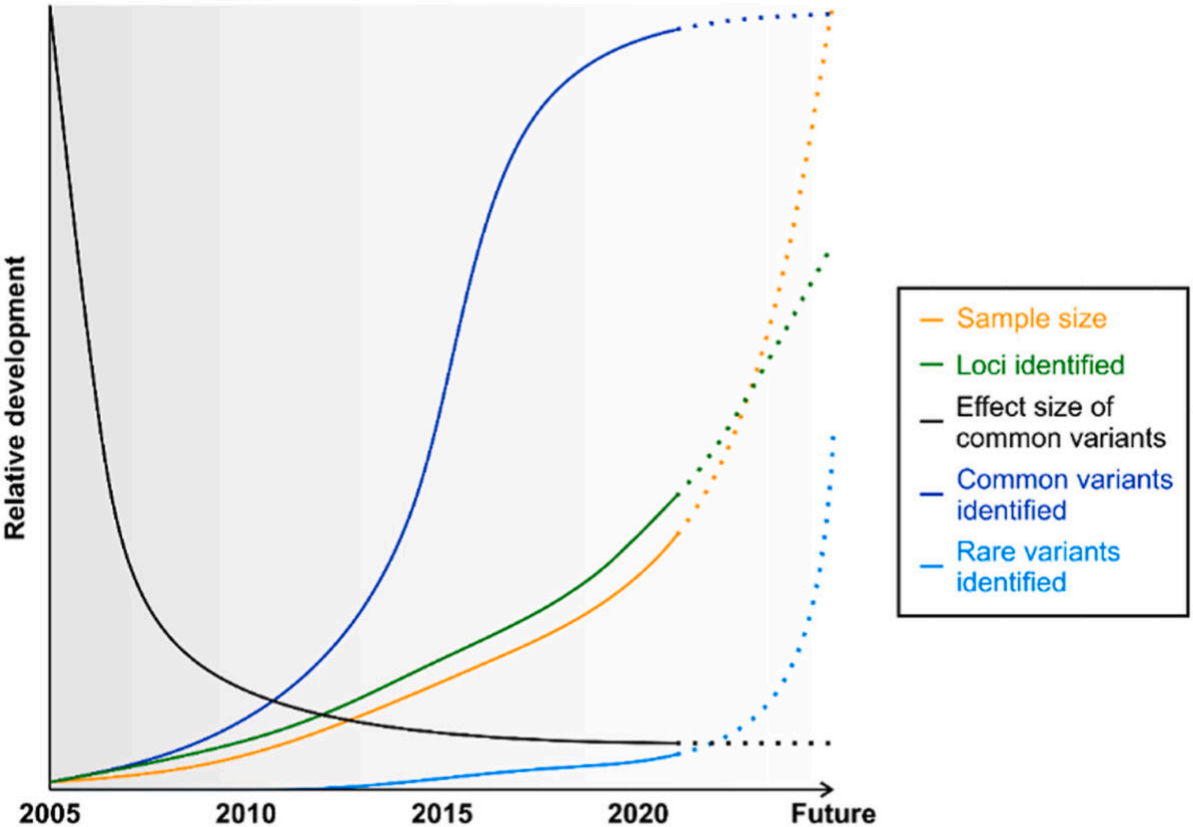
Looking back facilitates a look forward to appreciate key elements in GWAS advances of aAMD research. Sample sizes (orange) of GWAS studies reveal an exponential growth, enabling the detection of additional AMD-associated loci (green), while effect sizes (black) of common variants are predicted to decrease sharply in the next few years. In addition, the number of common variants identified (dark blue) rose steeply from 2011 onwards due to the use of genotype imputation (Yu et al., 2011). The next significant transformation will take place with the introduction of whole genome sequencing (WGS), which, in combination with increasing sample sizes, will allow the unbiased identification of numerous rare genetic variants associated with the disease (light blue).

There are opportunities and challenges interpreting data obtained from GWAS. A quick glance at the history of GWAS chronology in AMD research began with the pioneering work by Klein and colleagues (Klein et al., 2005), who illustrated the complexity of this methodology and its ability to provide meaningful information (Fig. 1). Despite a small sample size of only 96 AMD cases, it highlighted the genome-wide significance of the AMD-associated complement factor H (*CFH*) locus (Klein et al., 2005). This was the first demonstration of the value of GWAS, accepted by the field in part because it was independently validated by three other orthogonal study designs (Edwards et al., 2005; Hageman et al., 2005; Haines et al., 2005). From this initial study, research utilizing GWAS has evolved in many ways. Not only have sample sizes increased, but also the coverage of interrogated genetic variants across the human genome. The latter was made possible through markedly improved hardware for high throughput technologies, and refined algorithms such as genotype imputation. For example, genotype imputation allowed an estimation of association for over twelve million genetic variants using 800.000 genotyped variants as a proxy (Yu et al., 2011; Fritsche et al., 2013, 2016). These technical advances along with reduced genotyping costs led to the detection of common AMD-associated loci and variants, with decreasing effect sizes (Fritsche et al., 2016) (Fig. 1). Currently, common genetic variants located at 34 distinct loci are known to be associated with AMD, and as in other complex traits these variants are almost all in non-coding regions of the genome.

In addition to common alleles, the importance of rare genetic variants in complex diseases is well recognized and important due to their individual strong effect sizes on phenotypic traits (McClellan and King, 2010). Although individually rare variants confer small contributions to the overall inherited susceptibility of a disease, their identification allows the discovery of novel disease genes whose association may have stayed concealed if captured merely by the small effect size of its common alleles. In AMD, rare variants were identified in protein-coding regions of *CFH*, *CFI*, *C3*, *C9*, *TIMP3* and *SLC16A8* (Raychaudhuri et al., 2011; Helgason et al., 2013; Seddon et al., 2013; van de Ven et al., 2013; Zhan et al., 2013; Fritsche et al., 2016) and helped to establish the immediate role of the complement pathway in AMD pathogenesis.

Following the active phase of AMD-GWAS investigations between 2010 and 2016 (Chen et al., 2010; Neale et al., 2010; Yu et al., 2011; Fritsche et al., 2013, 2016), emphasis was placed on strategies to functionally evaluated AMD loci (Strunz et al., 2020b). Remarkably, of 703 articles citing recent GWAS (Fritsche et al., 2013, 2016), only 19% reported original experimental work focused on biological mechanisms underlying the GWAS associations. To date, of the 16 novel AMD loci reported in 2016, 13 have not been analyzed further (Strunz et al., 2020b). This could be ascribed in part to small effect sizes and possibly a preferential location of associated variants in non-coding regions of the genome. Nonetheless, advanced and high-throughput functional approaches are necessary to bridge from association studies into functional understanding of disease biology.

Omics emphasizes to generate large-scale data sets that lead to novel insight into structure and function of a cell, tissue, organ or organism. To fully integrate omics in the interpretation of GWAS data, a variety of quantitative trait locus (QTL) studies have been reported (Adriaens and Bezzina, 2018; Zheng et al., 2020), which describe the effect of one or more genetic variants on the levels of various types of molecules (e.g. RNA, DNA methylation, protein). Currently, QTL studies are exploring gene expression (eQTL), pre-mRNA splicing (sQTL), DNA methylation (mQTL), and protein expression (pQTL). GWAS data can directly be linked to QTL study results, thus greatly simplifying the identification of disease-relevant regulation of gene expression or mRNA splicing events (Giambartolomei et al., 2014). To this end, QTL-based omics tools appear to be an excellent starting point to bridge the gap between an association signal and its underlying disease-related molecular processes.

Also, omics provides new technologies that can be used to strengthen the power of established disease models. The characterization of disease-relevant cells or tissue, for example, is experiencing enormous advances due to single cell sequencing technology (Lukowski et al., 2019; Menon et al., 2019; Voigt et al., 2019b, 2021; Cowan et al., 2020). In addition, omics can hasten the identification of new biomarkers by analyzing the protein profiles of patient-derived tissues or blood samples empowering further Mendelian randomization studies (Grassmann et al., 2017; Porcu et al., 2019; Kiel et al., 2020). Those methods allow to conduct a so called “nature’s randomized trial” (Thanassoulis and O’Donnell, 2009) and have the potential to show causality of associated processes in the absence of strong pleiotropy. As such, many new perspectives have become available in recent years to investigate the genetic basis of complex diseases.

Regarding future developments, large population studies such as the UK Biobank (Sudlow et al., 2015) or the Million Veteran Program (Gaziano et al., 2016) will build on previous AMD research and increase the number of patients to well over 100,000 diseased probands. This increase (“the more the better”) will be accompanied by a significant rise in associated loci, but the effect sizes of these new loci are expected to be small (Fig. 1). Additionally, highly promising novel data from whole genome sequencing (WGS) of large AMD patient cohorts may identify rare variants, that are more likely functional, including small insertions/deletions or copy number variations. The inclusion of diverse ancestries with different distributions of common and rare variants, will facilitate fine-mapping and permit a more precise identification of AMD causal variants (Peterson et al., 2019). Mendelian randomization methods will allow exploration of causality for some genetic loci and the ability to determine if certain biomarkers (such as measurements in blood and non-invasive imaging) can be used as surrogates for efficacy of AMD therapies. The large GWAS studies from multiple populations and for other related medical conditions can serve in these types of studies to help advance our approaches to AMD.

Our current view on the architecture of late-stage AMD genetics is based on GWAS findings generated over the last 16 years. Currently, our knowledge on the genes or pathways involved is limited, nor do we yet sufficiently understand the underlying mechanisms of disease development. This is troublesome in the view of targeted therapies that are urgently needed, while their development depends on knowledge of the disease mechanisms. A broad omics approach is an appropriate strategy to close the gap between disease and its molecular understanding by linking association and function. Therefore, integration of omcis with GWAS is a necessary move in the post-GWAS era.

## 3. Single-cell RNA sequencing: insights into retinal health and disease

There are several motivations for understanding gene expression in human ocular tissues. A better understanding of the abundance and types of mRNA molecules in different cells and tissues can provide basic knowledge into the biology of cells in normal physiology, as well as in relation to age, disease status, and genotype; it can provide important baseline data for what constitutes a “normal” cell to guide the generation of stem cells for disease modeling and transplantation; it can provide new information about targetable pathways that are altered in diseases such as AMD; it can help answer where GWAS candidate genes are expressed; and it can improve our understanding of the cell types that are relevant for AMD risk.

The major sites of cellular degeneration in AMD (the neural retina and retinal pigment epithelium (RPE)/choroid) are complex tissues composed of numerous cell types. Previous gene expression studies of the neural retina and choroid relied on the analysis of mRNA prepared in bulk, where multiple cell types were lysed in aggregate and each contributed a fraction to an RNA pool that was converted to a complementary DNA (cDNA) library and evaluated (historically using differential display, serial analysis of gene expression, microarrays, and more recently RNA sequencing). These approaches, while powerful, can be biased by variation in cellular proportions between samples; for example, a higher proportion of ganglion cell-contributed RNA will be observed in the macular neural retina compared to extra-macular regions. Recent advances in microfluidics and next generation sequencing have been powerfully combined to study gene expression at single-cell resolution, and the commercialization of this technology has resulted in its widespread application across fields of medicine. Briefly, tissues are enzymatically dissociated into a solution of singlet cells as rapidly after collection as possible to minimize postmortem degradation of macromolecules. Cells are quantified and—using microfluidics—fused one to one with a droplet containing all the reagents required to generate a cDNA library from that particular cell. In this way, many thousands of cells are converted into thousands of independent cDNA libraries that can be sequenced together. The presence of unique nucleotide barcodes in each sample allows the investigators to know exactly which sequencing read pertains to which cell, and from which sample. Reads from each library are mapped to genes, and cells are then classified into known populations of cell types. RNA sequencing can be performed at a single *cell* level (single-cell RNA sequencing), or at a single *nucleus* level (single-nucleus RNAseq, snRNAseq)The nucleus contains reduced mRNA as compared to the whole cell, however, a significant advantage of snRNAseq is the ability to measure expression levels in frozen samples, which has opened the door to profile banked post-mortem human tissues.

Since its emergence, multiple groups have used scRNAseq and snRNAseq to study ocular tissues in human neural retina, RPE and choroid; from both central and peripheral regions including segregation of the retina into foveal and parafoveal regions; and from control and AMD donors (Lukowski et al., 2019; Voigt et al., 2019a, 2019b, 2021; Orozco et al., 2020; Yan et al., 2020; Lyu et al., 2021). These studies have for example provided insights into the molecular distinctiveness of cones from different topographic regions. Foveal cones are structurally distinct from peripheral cones. In the fovea, cones have very long axons that comprise Henle’s layer of the neural retina and lack the teardrop shaped inner segments that are characteristic of peripheral cones (and that were noted by Cajal, (Ramon y Cajal, 1972)). Instead, these central-most cones possess columnar inner segments and relatively long outer segments. In addition to their anatomical uniqueness, foveal cones were also found to differ at the molecular level from peripheral cones, harboring higher expression of the mRNA for the synaptic protein *RIMS2* and reduced expression of the mRNA encoding the carotenoid cleaving enzyme *BCO2*, a difference which may contribute to the spatial distribution of macular pigment (Voigt et al., 2019b).

**Fig 2.**
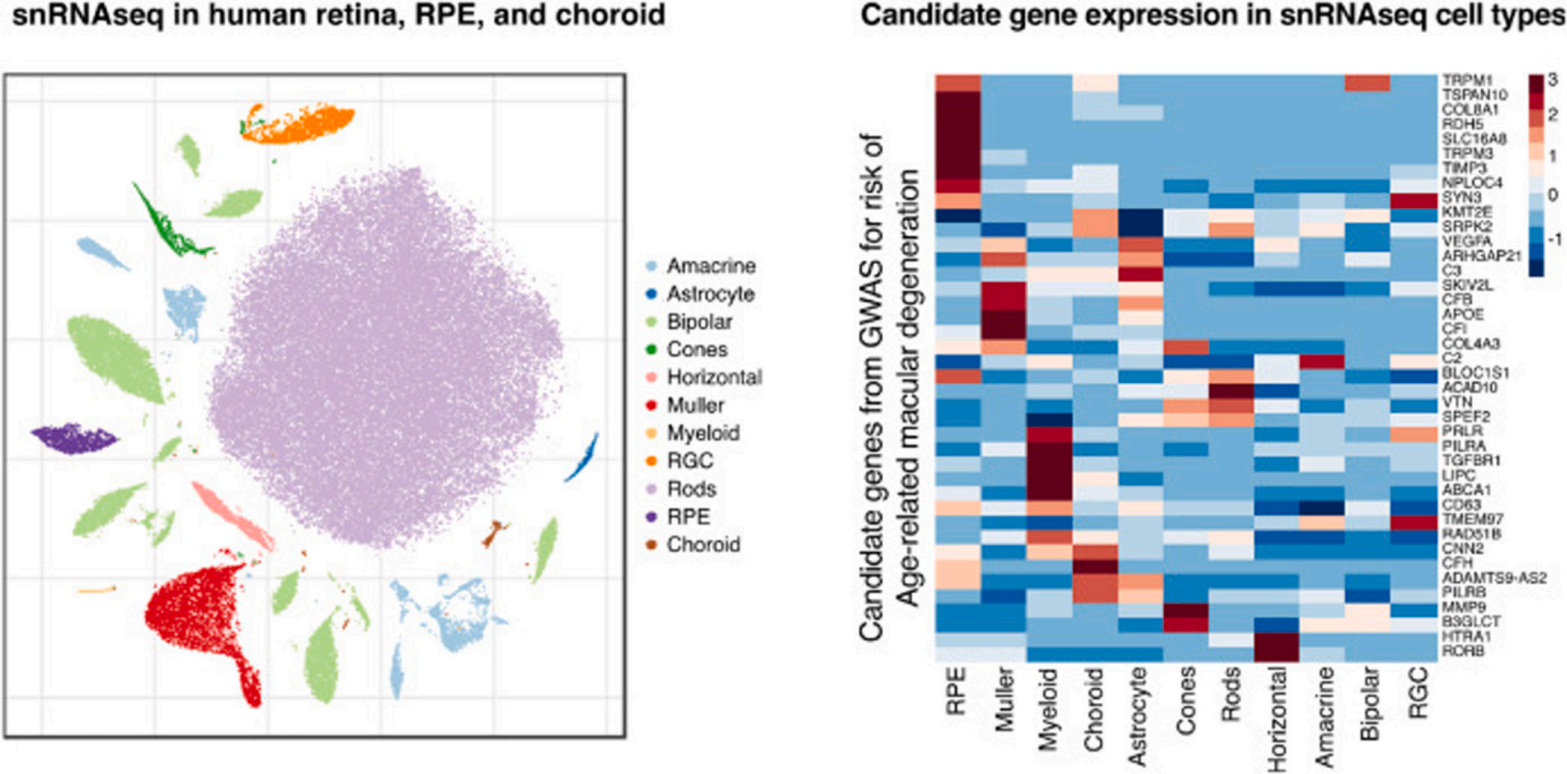
(A) Single-nucleus RNAseq profiling of human ocular tissue identifies all major cell types in the retina, RPE, and choroid. (B) Expression from snRNAseq shows that the majority of candidate genes from the AMD GWAS are expressed in non-neuronal cells such as RPE, glia, myeloid, and choroidal cells.

Single-cell studies of the retina have also been explored in rodent and primate model organisms (Macosko et al., 2015; Shekhar et al., 2016; Clark et al., 2019; Peng et al., 2019; Tran et al., 2019), and *in vitro* retinal organoids (Sridhar et al., 2020). Orozco, Chen et al. used snRNAseq to create an atlas of expression profiles from aged human eyes frozen post-mortem (Orozco et al., 2020). The resulting dataset is comprised of over 100,000 cells across dozens of ocular cell types (Fig. 2A), including retinal neuronal cells such as amacrine, bipolar, cone and rod photoreceptors, and retinal ganglion cells (RGC), as well as Müller and astrocyte glia, RPE cells, myeloid cells, and choroidal cells such as endothelial, fibroblast, melanocyte, pericyte and Schwann cells. The scale of the study allowed fine cell subtype resolution, where resolution was directly proportional to the number of cells identified for a given cell type. Since this publication, two studies of comparable scale (Yan et al., 2020; Lyu et al., 2021) were published using scRNAseq in human adult retinas. While these studies are focused on the neural retinal cells and do not contain RPE or choroidal cells, the use of single-*cell* RNAseq instead of single-*nucleus* RNAseq makes them a valuable complement to the findings published by Orozco, Chen et al. (Orozco et al., 2020). Furthermore, Yan et al. (2020) used cell sorting to enrich for cell types with a high degree of heterogeneity, resulting in finer cell subtype resolution for non-photoreceptor cell types such as RGCs.

In addition to neural retina, our understanding of the choroidal endothelium has also been improved by the application of scRNAseq. This is especially relevant in light of recent discoveries that choriocapillaris damage is a prominent and early disease feature of AMD (Mullins et al., 2011; Lutty et al., 2020). In contrast to the notion that the choroid is predominantly comprised of blood vessels, over a dozen distinct choroidal cell types can be readily distinguished, with endothelial cells representing about 15% of all choroidal cells in the macula. Moreover, arterial, vein, and choriocapillaris endothelial cells can be discriminated (Supplementary Fig. 1), and potential genotype- and disease-related alterations in gene expression assessed in each cell type. This led to the identification of *RGCC* (a complement activated gene) as exhibiting increased expression in the macular (compared to peripheral) choriocapillaris and in neovascular choroidal endothelial cells (Voigt et al., 2019a). An interactive web-based resource, Spectacle, was developed by the University of Iowa, which provides an easily accessible platform to explore previously published single-cell RNA sequencing data from ocular studies (Voigt et al., 2020). Spectacle is freely available via the web at https://singlecell-eye.org, making it easy to quickly identify cell types that express a gene of interest.

Single cell sequencing studies of the retina, RPE and choroid determined which cell types are enriched for expression of genes in or near AMD GWAS loci (Menon et al., 2019; Cowan et al., 2020; Orozco et al., 2020). In a scRNAseq study of the neural retina, AMD-associated genes were enriched in glial cells, vascular cells and cone photoreceptors (Menon et al., 2019). In a scRNAseq study of foveal and peripheral neural retina, RPE and choroid, AMD-associated genes were expressed in vascular endothelial cells, fibroblasts, monocytes, RPE, bipolar cells, and horizontal cells (Cowan et al., 2020). In a snRNAseq study of the neural retina, RPE, and choroid (Orozco et al., 2020), the majority of candidate genes identified through GWAS for AMD risk are expressed in non-neuronal cells such as RPE, glia, myeloid and choroidal cells (Fig. 2B), with the largest proportion of genes expressed in RPE cells (38%). Indeed, putative causal genes in GWAS loci for AMD risk, identified through colocalization of GWAS and eQTL, were found to be driven by expression in the RPE (e.g. *TSPAN10, TRPM1*). These studies strengthen the hypothesis that AMD risk largely originates in the RPE and choroid, and highlight that many different retinal and choroidal cell types contribute to the pathogenesis of AMD.

While single cell sequencing analysis of tissues is very powerful, it does have challenges. Like other technologies for analyzing RNA, short death-to-preservation times (certainly on the order of single digit hours) is important in preserving macromolecules. For scRNAseq, the dissociation of tissues into single cells needs to be optimized, and the requirements for collecting individual cells in loose connective tissue differ from those of cells embedded in dense extracellular matrix or adhered to each other with desmosomes. In some cases, to optimize the analysis of a critically important cell type that is underrepresented (e.g., choroidal endothelial cells), the enzymatic digestion must be consistent with maintaining the antigenicity of cell surface proteins (such as PECAM1). Finally, a prominent hurdle for AMD research is the scarcity of well-phenotyped and properly preserved human eye frozen tissue banks akin to the established brain banks. AMD disease-specific molecular analyses are still scarce (Voigt et al., 2019a; Lyu et al., 2021), and expanded characterization of AMD ocular tissues at the single cell level is needed to further elucidate disease mechanisms. Despite these challenges, studies on non-diseased human retina, RPE and choroid and animal models provide a valuable resource for exploring and understanding specific cell type expression in ocular tissues, and provide context to target these cell types for the development of new therapies (Menon et al., 2019). While single cell sequencing studies have identified altered expression in RPE, glia, myeloid and choroidal cells, it is important to realize that these changes may alter other cell types in mediating risk or protection from AMD.

## 4. Prioritizing causal genes for AMD using eQTL

GWAS can leverage naturally occurring genetic variation in human populations, that differ between disease cases and controls, in order to identify genomic regions associated with disease risk. While GWAS can identify genomic regions associated with a phenotype of interest, they do not provide sufficient resolution to pinpoint the specific causal genes underlying the association. On the one hand, each locus typically harbors several candidate variants and thus genes in linkage disequilibrium; on the other hand, a large proportion of the GWAS SNPs associated with a phenotype tend to fall in non-coding regions, which are believed to harbor regulatory regions for both nearby and distal genes. Hence, a crucial issue in AMD etiology is to elucidate the causal variations underlying the genetic loci associated with disease. To accomplish this, systems genomics employs a multi-faceted approach exploring chromatin accessibility, tissue and cell type expression, protein levels, and the integration between genetics and molecular phenotypes.

**Fig 3.**
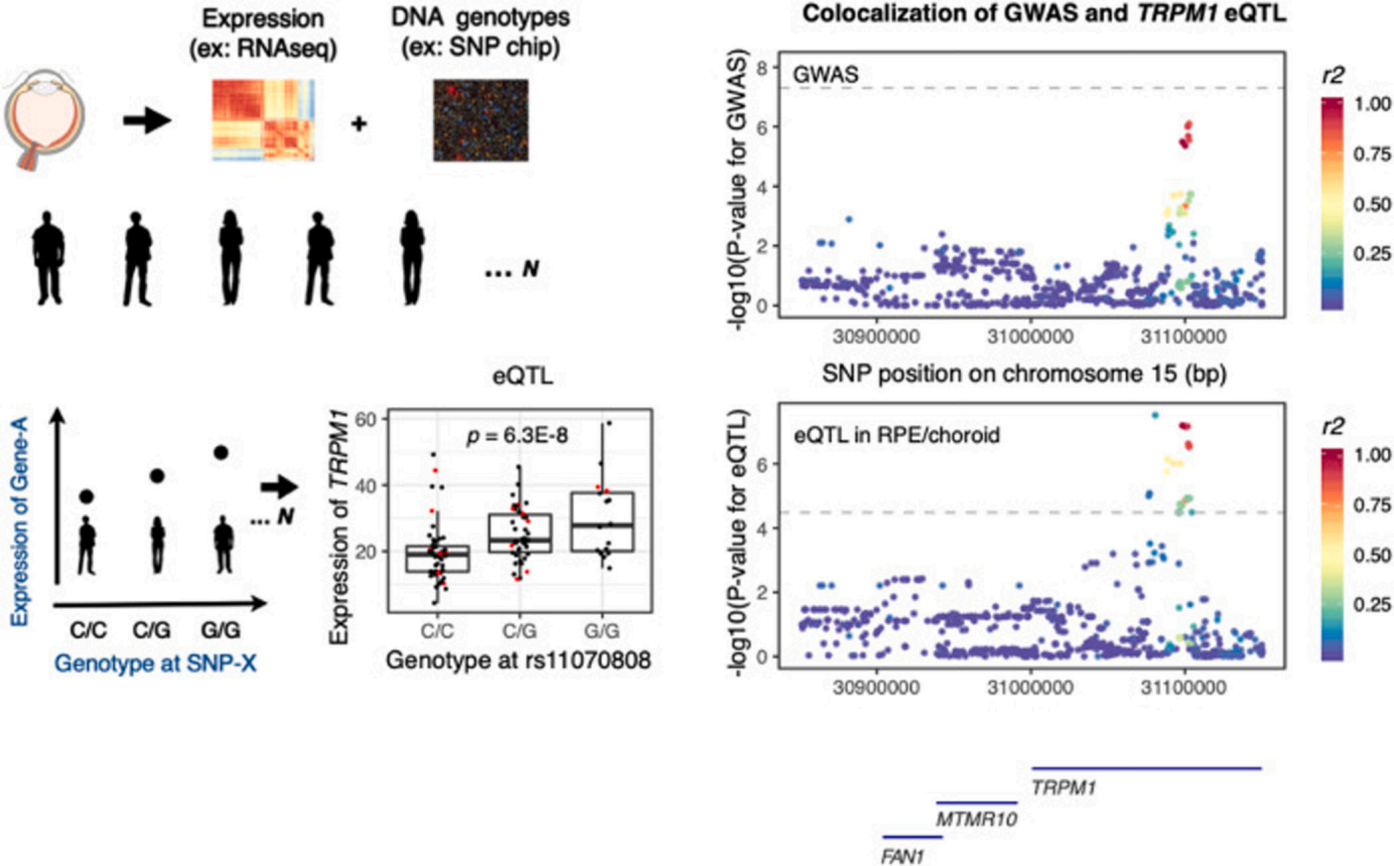
(A) Approach for estimating eQTL, using ocular tissue and expression of *TRPM1* as an example. (B) Colocalization between GWAS statistics for risk of AMD, and eQTL in the RPE/choroid tissue suggest *TRPM1* is the causal gene at this locus.

Expression QTL (eQTL) can be a powerful tool for prioritizing and discovering causal genes underlying genetic associations, particularly if these studies are performed in the cell types and tissues that are relevant to the disease/phenotype biology. In eQTL studies, scientists profile expression levels and DNA genotypes across the genome in a cohort of individuals. For each gene, relationships are assessed between gene expression levels and genotypes for all individuals in the cohort, using SNPs physically located near the gene (typically within 1 Mb). These relationships can formally be tested using linear regression to identify genetic loci driving expression levels, or eQTL (Fig. 3A). Putative causal genes can be prioritized in GWAS loci by searching for genes with eQTL in the region identified by GWAS. Specifically, the GWAS signal and the eQTL should colocalize (Giambartolomei et al., 2014), *i.e.* there should be evidence that the same SNPs that are associated with a phenotype in the GWAS are also associated with expression levels of a given gene.

To date, three studies have employed eQTL from retinal, RPE and choroidal tissues to elucidate AMD GWAS loci (Ratnapriya et al., 2019; Orozco et al., 2020; Strunz et al., 2020a). Orozco et al. performed eQTL analysis in a cohort of 121 human donor eyes, using bulk tissue RNAseq from four regions of the eye: the neural retina and RPE/choroid, each derived from either macular or extra-macular regions (Orozco et al., 2020). Using colocalization (Fig. 3B), they identified 15 genes in 13 AMD risk loci that show a genetic overlap between gene regulation and AMD risk. Five of those genes were different from the candidate gene originally assigned based on physical proximity to the genetic variants associated in the GWAS. These genes highlight inflammation, complement, angiogenesis, extracellular matrix, vesicle trafficking, and pigmentation pathways. Similarly, Ratnapriya et al. performed eQTL analyses in bulk tissue from peripheral neural retina in a cohort of 453 donor eyes, and identified putative causal genes in 6 GWAS loci for AMD (Ratnapriya et al., 2019). Evidence in 5 of these loci highlight the same genes in both ocular eQTL studies (*B3GLCT, BLOC1S1, CFI, PILRA/PILRB* and *TMEM199*), one putative causal gene was uniquely identified by Ratnapriya et al. in the neural retina eQTL (*SH2B3B*), and eight genes were uniquely identified by Orozco et al. using eQTL from RPE/choroid (*BAIAP2L2, COL4A3, HTRA1, RDH5, TNFRSF10A, TRPM1, TSPAN10*), and the neural retina (*BCAR1, SLC12A5-AS1*). A recent analysis of 311 retina samples identified 403,151 significant eQTL variants that regulate 3007 genes (Strunz et al., 2020a). When correlating these eQTLs with AMD GWAS loci, the eQTLs at the *PILRA/PILRB* genes identified in the other two eQTL studies were confirmed, and 5 additional retina eQTLs at the AMD loci were identified.

A recent transcriptome-wide association study was performed using transcriptomics datasets of 27 tissues to predict the effects of AMD-associated genetic variants on gene expression outside of the eye. This study identified 88 genes in the AMD-associated loci that were significantly associated with AMD in a least one tissue (Strunz et al., 2020c). Four of these genes showed remarkably large effect sizes: *CFHR1*, *CFHR3,* and *CFHR4* showed higher gene expression in cases compared to controls with the largest effect in liver, while *ARMS2* showed lower gene expression in cases compared to controls with the strongest effect in testis. Finally, a study of plasma protein QTL (pQTL) identified overlaps between AMD GWAS loci and pQTL for *APOE*, *CFH*, and *CFB* (Sun et al., 2018). These findings are consistent with the multifactorial nature of AMD risk, where both ocular and systemic gene expression are believed to contribute to disease risk, and suggest that studies using eQTL and pQTL across different cell types and tissues can provide complementary evidence for causal genes and pathways driving AMD.

## 5. Effects of AMD-associated genetic variants on complement proteins in the circulation

GWAS have pointed towards an important role for the complement system in AMD. Of 52 genetic variants that were independently associated with AMD by GWAS, 19 localized in or near genes of the complement system (Fritsche et al., 2016; de Jong et al., 2021). One of the strongest AMD risk associations in the complement system was reported for the common p.Tyr402His (rs1061170) variant in the complement factor H (*CFH*) gene (Edwards et al., 2005; Hageman et al., 2005; Haines et al., 2005; Klein et al., 2005). Since then, additional genetic variation at the *CFH* locus has been reported to confer either increased risk (rs570618, rs121913059, rs187328863, rs35292876, rs191281603) or a decreased risk (rs10922109, rs148553336, rs61818925) for AMD (Supplementary Fig. 2) (Fritsche et al., 2016). Variant rs570618 is located in an intron of the *CFH* gene, but is in high linkage disequilibrium (meaning that it is inherited together) with the p.Tyr402His variant. The rs121913059 variant is a rare coding variant leading to an amino acid change (p.Arg1210Cys) in Factor H (FH), which confers a strong risk for AMD with an odds ratio of 47.6 (conditioned for all AMD-associated variants). In addition, a common deletion spanning the *CFHR3* and *CFHR1* genes is associated with a decreased risk for AMD (Hughes et al., 2006). A recent analysis of haplotypes and diplotypes at the *CFH* locus suggested that the strong protective effects at this locus may originate from the *CFH* coding variant p.Ile62Val and the *CFHR3/CFHR1* deletion (Pappas et al., 2021).

In addition to genetic variation at the *CFH* locus, variants in or near the complement factor B (*CFB*), complement component C3 (*C3*), complement component C9 (*C9*), complement factor I (*CFI*) and vitronectin (*VTN*) genes have been associated with AMD (Gold et al., 2006; Yates et al., 2007; Fritsche et al., 2016). These variants include intergenic, intronic and coding variants that either confer an increased or decreased risk for AMD. Coding variants in these genes include the variants p. Gly119Arg (rs141853578) in *CFI*, p.Arg102Gly (rs2230199) and p. Lys155Gln (rs147859257) in *C3*, and Pro167Ser (rs34882957) in *C9*, which are associated with an increased risk of AMD. In addition, two coding variants in the *CFB* gene, p.Leu9His (rs4151667) and p.Arg32Gln (rs641153), are associated with a decreased risk for AMD.

Using a gene-based approach, rare coding variants in the *CFH* and *CFI* genes were found more frequently in AMD patients compared to controls (Fritsche et al., 2016). In addition, targeted sequencing of selected complement genes further lead to the identification of rare coding variants in the *CFH*, *CFI*, *C3* and *C9* genes (Raychaudhuri et al., 2011; Helgason et al., 2013; Seddon et al., 2013; van de Ven et al., 2013; Zhan et al., 2013). Although their overall frequency in the population is limited, carrying a rare variant substantially increases the risk for AMD in individual patients (de Breuk et al., 2021a). Overall, the genetic architecture of AMD is composed of common and rare variants, where common variants generally have limited effects sizes (with the exception of the p.Tyr402His variant in *CFH*), while rare variants can have very large effects on the development of AMD (Fritsche et al., 2016; Geerlings et al., 2017).

Single cell sequencing studies have shown that many complement components are expressed locally in neural retina, RPE, and choroidal cell types, but most are highly expressed in the liver. Since they are secreted in the systemic circulation, their levels can be measured in serum or plasma samples. Several studies have used measurements of complement proteins in the circulation to determine the functional effect of genetic variants in the complement system. The AMD-protective p.Leu9His variant in *CFB* was associated with reduced circulating systemic factor B (FB) levels (Hecker et al., 2010; Smailhodzic et al., 2012). Recent studies reported the effects of genetic variants at the *CFH* locus on systemic factor H (FH) and factor H related (FHR-1, FHR-2, FHR-3, FHR-4 and FHR-5) protein levels. FHR-4 levels were strongly associated with variants at the *CFH* locus, where AMD risk-conferring variants (rs570618, rs187328863) were associated with increased FHR-4 levels, while protective variants (rs10922109, rs61818925) were associated with decreased FHR-4 levels (Cipriani et al., 2020). In two recent studies evaluating the systemic levels of all five FHR proteins (Cipriani et al., 2021; Lores-Motta et al., 2021), common AMD genetic variants and haplotypes at the *CFH* locus strongly associated with FHR-1, FHR-2, FHR-3, FHR-4 and FHR-5 protein concentrations, whereas the association with FH concentrations was limited (Supplementary Fig. 2). Even stronger effects on FHR concentrations were reported for haplotypes constructed for variants across the *CFH* locus, compared to the individuals genotypes. AMD-protective haplotypes H2, H3, H4, H5 and H7 at the *CFH* locus were generally associated with reduced FHR concentrations. The strongest effects were identified for the H2 AMD-protective haplotype, which associated with reduced FHR-2 and FHR-4 concentrations. The AMD-protective haplotypes H3 and H7 carry the *CFHR3-CFHR1* deletion, and, as expected, were associated with reduced FHR-1 and FHR-3 concentrations (Lores-Motta et al., 2021). Mendelian randomization analyses supported a causal role of the FHR-1, FHR-2, FHR-4 and FHR-5 proteins in AMD (Cipriani et al., 2021). In addition, low-frequency protein-altering variants in the *CFHR2* and *CFHR5* genes, which were associated with a decreased risk for AMD, led to reduced or absent FHR-2 and FHR-5 concentrations, respectively (Lores-Motta et al., 2021). As a recent analysis suggested that the strong protective effects at this locus may originate from the *CFH* coding variant p.Ile62Val and the *CFHR3/CFHR1* deletion (Pappas et al., 2021), further experimental work is needed to determine the role of the *CFH* p.Ile62Val variant on decreased FHR levels.

The effects of individual rare coding variants on levels of complement components, in particular in the *CFH* and *CFI* genes, have been established using serum and plasma of carriers of these variants. To date, 160 coding variants in the *CFH* gene and 110 coding variants in the *CFI* gene have been reported in AMD, leading to either nonsense, frameshift, or missense changes in the FH and factor I (FI) proteins, respectively (de Jong et al., 2021). A recent study measured systemic FH levels in plasma of 252 carriers of 64 different rare and low frequency variant in the *CFH* gene (de Jong et al., 2022). For eight variants, significantly reduced FH levels were identifed in plasma of carriers, and for 11 variants FH plasma levels were below the normal range in at least a single carrier. In a grouped analysis, carriers of missense, nonsense and frameshift variants showed significantly reduced FH plasma levels compared to non-carrier cases and non-carrier controls. In addition, C3bBbP levels were measured in these 252 carriers as a marker for complement activation. C3bBbP levels were signficiantly elevated in carriers of three *CFH* variants, and for nine variants C3bBbP levels in a single carrier were above the normal range. This indicates that FH function may be impaired by these *CFH* variants.

Several recent studies demonstrated that FI plasma levels are commonly reduced in carriers of rare *CFI* variants (Kavanagh et al., 2015; de Jong et al., 2020; Hallam et al., 2020). More than one-third of AMD patients carrying a rare *CFI* variant had FI levels in the fifth percentile, and low FI levels in serum present a higher risk of developing AMD (Hallam et al., 2020). The common effect of rare *CFI* variants on reduced FI concentrations was confirmed by recombinant FI expression levels for 126 rare coding variants. Of these 126 variants 68 (54%) resulted in significantly reduced FI expression of mutant protein in supernatant compared to wildtype protein (de Jong et al., 2020). Therefore, more than half of rare *CFI* variants lead to reduced FI levels. For *CFI* variants that have normal FI levels, it was demonstrated that they may lead to reduced FI function by measuring systemic complement activation products, C3b and iC3b (Java et al., 2020).

In conclusion, complement measurements in the systemic circulation have aided in understanding the effect of genetic variants in the complement system in AMD (de Jong et al., 2021). The protective p.Leu9His variant in *CFB* was associated with reduced FB levels in plasma. Common genetic variants and haplotypes at the *CFH* locus were strongly associated with FHR levels, pointing towards a role for the FHR proteins in AMD. Strong protective effects at the *CFH* locus were associated with reduced FHR protein concentrations, and have been suggested to alleviate risk at the *CFH* and *ARMS2/HTRA1* loci (Pappas et al., 2021). Rare coding *CFH* and *CFI* variants lead to reduced FH or FI levels or function. It remains to be determined whether the altered complement levels in the systemic circulation are just a reflection of the effect of AMD-associated genetic variants, which exert their effect throughout the body, or whether the complement factors that circulate in the blood also reach the retina and contribute to AMD manifestation. Although many complement proteins are produced in the liver, single cell sequencing studies support local expression of complement genes in specific retinal, RPE and choroidal cell types. Since eQTL and TWAS studies showed that many AMD-associated genes may exert their effect in cells outside of the retina (Strunz et al., 2020c), pQTL studies - extending to proteins outside of the complement system - in plasma and serum samples may aid in understanding the effect of genetic variants and pinpointing causal genes in AMD.

## 6. Human induced pluripotent stem cell (iPSC)-derived RPE: a model system for prioritizing and functionally characterizing causal variants at AMD risk loci

To gain more detailed knowledge with respect to the molecular mechanisms of AMD and on the effect of AMD-associated genetic variants, cellular-based models can be employed. Since the RPE is a critical cell type inAMD pathogenesis, the RPE cell lines ARPE19 and hTERT RPE1 have often been used to study AMD-related processes. Recently, the use of RPE cells differentiated from induced pluripotent stem cells (iPSC) became more popular in AMD research. The advantage of iPSC-RPE cells over using immortalized cell lines ARPE19 and hTERT1 is that such cells can be generated from AMD patients, carrying relevant AMD-associated genotypes. Furthermore, iPSC-RPE cells in culture have been shown to display key physical, gene expression and functional properties similar to human RPE cells in vivo (Dalvi et al., 2019). This makes these cells a better model to study RPE-specific functions, such as phagocytosis of photoreceptor outer segments (Westenskow et al., 2012). An important disease characteristic of AMD is the formation of sub-RPE drusen and detachments. Interestingly, drusen-like deposits also appear in long-term cultures of RPE cells on membranes, as shown in human fetal RPE (Johnson et al., 2011; Fernandez-Godino and Pierce, 2018; Chen et al., 2020), primary RPE cells (Pilgrim et al., 2017) and iPSC-RPE (Galloway et al., 2017), making these cell types interesting to model AMD disease characteristics.

To investigate the utility of iPSC-RPE as a model system to prioritize and functionally characterize causal variants at AMD risk loci, iPSC-RPE were derived from six female subjects of multiple ethnicities (Caucasian, Asian, and African American; AMD disease status unreported) (Panopoulos et al., 2017; Smith et al., 2019). These cells exhibited morphological and molecular characteristics similar to those of native RPE. Across all iPSC-RPE samples, virtually all cells were hexagonal in shape and strongly pigmented. Moreover, a high fraction of the cells were positive for zonula occludens 1 (ZO-1) and/or microphthalmia-associated transcription factor (MITF) (ZO-1+ mean = 93.8%; MITF mean = 99.0%; and double positive = 91.5%). Two of the iPSC-RPE samples were also examined for characteristic RPE marker proteins, and membrane expression of ZO-1 and bestrophin 1 (BEST1) and nuclear expression of MITF were observed. Thus, the derived iPSC-RPE displayed morphological characteristics and expressed marker genes of naïve RPE.

**Fig 4.**
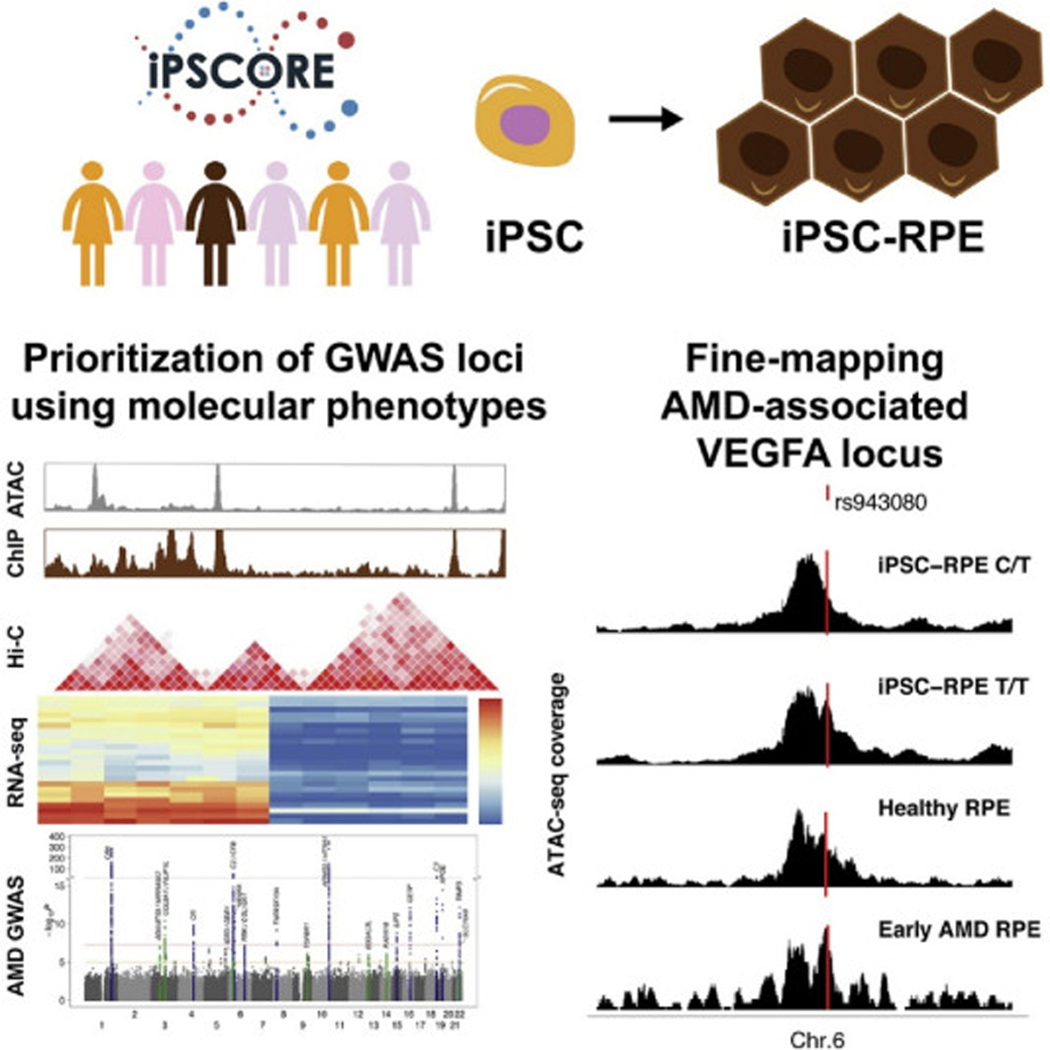
iPSC-RPE can be used to prioritize and functionally characterize causal variants at AMD risk loci. iPSC-RPE from six subjects in the iPSCORE resource were generated and shown to have molecular and morphological characteristics similar to native RPE. We generated ATAC-seq, RNA-seq and H3K27ac ChIP-seq data from the iPSC-RPE. To fine map AMD risk loci we used fgwas to integrate these molecular phenotype data with AMD GWAS. rs943080 was identified as the probably causal variant in the *VEGFA* locus.

To investigate similarities in gene expression and the epigenome of iPSC-RPEs and naïve RPE, RNA sequencing data (RNA-seq), Assay for Transposase-Accessible Chromatin sequencing data (ATAC-seq), and histone acetylation (H3K27ac) chromatin immunoprecipitation sequencing data (ChIP-seq) were generated from the six iPSC-RPEs (Fig. 4) (Smith et al., 2019). Using principal component analyses, global gene expression in the six iPSC-RPEs, iPSCs, iPSC-derived cardiomyocytes, fetal RPE, adult RPE, adult neural retina, peripheral RPE-choroid-sclera and the APRE-19 cell line were examined. The iPSC-RPEs clustered with the fetal RPE samples while the adult RPE and peripheral RPE-choroid-sclera samples clustered together. The iPSC-RPE also expressed RPE signature genes, and the expression of these signature genes was similar to that of fetal RPE. To examine chromatin accessibility profiles of the iPSC-RPE, ATAC-seq data were generated and compared with ATAC-seq data from fetal RPE and adult RPE including individuals both with and without AMD. ATAC-seq peaks in all samples were enriched for transcription factor (TF) motifs important for RPE development (OCX2, CRX and SOX9). However, the iPSC-RPE samples were more similar in TF motif enrichment profiles to fetal RPE than adult RPE. Overall, these data show that iPSC-RPE and human fetal RPE share highly similar gene expression and enriched transcription factor motif profiles.

The majority of AMD GWAS risk loci are located in non-coding regions and presumably the causal underlying variants are regulatory in nature; therefore, the study aimed to determine if the regulatory regions in iPSC-RPE could be used to fine-map AMD causal variants (Smith et al., 2019). The results demonstrated that iPSC-RPE H3K27ac peaks and ATAC-seq regions as well as fetal RPE, adult AMD RPE, and adult healthy RPE ATAC-seq peaks were enriched for AMD genetic risk; additionally, exon regions and missense variants were enriched. A functional GWAS (FGWAS), a command line tool for integrating functional genomic information into a GWAS (Pickrell, 2014), was performed to fine map 32 AMD risk loci and identify potentially causal variants. Twenty of the FGWAS lead variants were classified based on their associations with molecular annotations: ten were distal regulatory variants, six were local regulatory (overlapping promoter), and four were missense coding variants. These results show that iPSC-RPE are a good model system for prioritizing and functionally characterizing causal variants at AMD risk loci.

A variant (rs943080) located in an ATAC-seq peak in the *VEGFA* locus was investigated further, as FGWAS indicated that it was a strong candidate for being the causal variant underlying the GWAS signal (Fig. 4) (Smith et al., 2019). Of the six subjects from whom iPSC-RPEs were derived, five were heterozygous for rs943080 (C/T) and one was homozygous for the risk allele (T/T). Across the five heterozygous individuals, the risk allele showed strong chromatin accessibility indicating that the regulatory element was more active. The five individuals heterozygous for rs943080 also showed significantly higher expression of *VEGFA* than the one homozygous risk sample, indicating that the risk allele drives decreased *VEGFA* expression in RPE. Further investigation of the locus showed that the altered expression levels were likely driven by decreased expression of a non-coding *VEGFA* transcript. These findings suggest that while the rs943080 risk allele is associated with an overall decreased level of *VEGFA* expression, it is likely mediated through the altered expression of a specific non-coding transcript.

This study provided insight into the *VEGFA* AMD GWAS locus with relatively few iPSC-RPE samples because by chance five individuals were heterozygous and one was homozygous for the risk variant (Smith et al., 2019). To gain insights into the other AMD risk loci, it is likely that samples from hundreds of individuals are needed. Because high-quality RPE samples are challenging to get from human cadavers and sample limitations may restrict molecular characterization, it may be better to utilize iPSC-RPE as a *model* system. These findings show that deriving iPSC-RPEs from hundreds of different individuals could lead to the identification and functional characterization of the causal variants in the vast majority of the AMD risk loci.

## 7. Synthetic haplotypes to understand AMD development

An ultimate goal of clinical genetics is to identify specific variants with a functional impact on a human phenotype of interest, such as the susceptibility of a given individual to AMD based on their genomic DNA sequence. “GWAS hits” have gone the distance in cases where the variants of interest are coding variants (cSNPs) but the vast majority of variants that define GWAS hits lie in relatively uncharted noncoding regions, both between (intergenic) and within (intronic) genes. The variants of interest typically lie in haplotype blocks of tens to hundreds of kilobases, along with 10s to hundreds of variants that are in LD with the index SNPs. Importantly, because they are noncoding, these GWAS hits are of smaller effects than are coding variants, contributing to the difficulty in pinning down the critical variants. Additionally, more than one clustered variant may be required to underlie a functional difference. Computational tools can help prioritize these variants based on overlap with regions of enhanced DNA accessibility, such as DNAse Hypersensitive site mapping, ATAC-Seq, or locations of enhancer-specific chromatin modifications. However, even with such stratification there are often tens or more variants in consideration.

Because of the fact that the variants represented in a haplotype are in strong LD with each other, i.e. strongly co-inherited, screening human populations only rarely turns up natural recombinants in populations under study. One approach being taken by the Boeke group which is intended to solve this problem is to produce synthetic DNAs called assemblons representing two forms of a human locus, typically representing risk and protective haplotypes. These are then either synthesized de novo, amplified from human gDNA of known genotypes, or sourced from a collection of bacterial artificial chromosomes (BACs) like the BACPAC Consortium, using yeast assembly to stitch together fragments of synthetic and/or natural DNA to directly build these 100+ kb molecules. Once these are assembled, they can be delivered to stem cells, either human or murine, to an engineered landing pad previously delivered using CRISPR, or similar tools. In our recently described BIG-IN method, we exploit a landing pad with a counterselectable marker flanked by heterotypic loxP sites which are also found at the extremities of the assemblon or payload DNA being delivered, which can be 140 kb or more (Brosh et al., 2021). These methods are streamlined to work in mouse embryonic stem cells, and can be deployed for a series of variant constructs so that they are always delivered to the same location and cells of identical genetic background. Further they can be selectively delivered to one allele so that copy number is controlled for. Once a phenotype, transcriptional or otherwise, is identified that varies with risk vs protective haplotype, it is possible do a “haplotype dissection” in order to identify the variant or variants responsible for the phenotype (Laurent et al., 2019a, 2019b). This haplotype dissection can be performed exploiting yeast homologous recombination in various ways to juggle the variants into a series of assemblons differing only in the constellation of variants each has. This strategy is illustrated in Supplementary Fig. 3.

Ultimately the stem cells can either be studied directly, or differentiated, for example into RPE-like cells. If the stem cells are murine, they can be turned into an animal and in that way studies of the organ itself can be performed, and importantly, even as a function of time in aging animals.

Another advantage of the synthetic approach is that it is possible to use recombination in yeast to engineer reporters into the assemblons, greatly simplifying the readouts of interest. For example, in many instances there may be two genes flanking the ncDNA of interest and it is not clear which gene is affected by the risk/protective haplotype. By engineering a GFP reporter into the C-terminus of one gene and an RFP gene into the C-terminus of the other, it is possible to assess expression of both genes at once; and one can even consider pooling of different ESC clones containing distinct haplotypes or combinatorial variants in this context.

## 8. Clinical trials for GWAS hits

Pinpointing causal genes and understanding disease mechanisms is crucial for the next step towards designing effective clinical trials. Several clinical trials targeting proteins encoded by AMD GWAS loci (*C3*, *CFB*, *CFI*, *CFH*, *ARMS2/HTRA1*) have been completed or are currently ongoing (de Jong et al., 2022). Two therapeutic strategies targeting the central component C3 have been performed (POT-4, Alcon Research; APL-2, Apellis) and one is currently in phase II (NGM621, NGM Biopharmaceuticals) for geographic atrophy (GA). A phase III clinical trial (OAKS) evaluating a synthetic cyclic peptide conjugated to a polyethylene glycol polymer that binds specifically to C3 and C3b (APL-2, pegcetacoplan) showed a 22% reduction in GA lesion growth, but the parallel DERBY study did not show consistent findings (https://investors.apellis.com/news-releases/news-release-details/apellis-announces-top-line-results-phase-3-derby-and-oaks). Ionis Pharmaceuticals is currently leading a phase II clinical trial in GA involving subcutaneous administration of an antisense inhibitor (IONIS-FB-LRx) targeting the *CFB* gene for systemic reduction of circulating FB levels. In addition, Gyroscope Therapeutics is leading an ongoing phase II clinical trial for subretinal AAV2-based *CFI* gene therapy (GT005) for supplementation of FI protein in the eye (de Jong et al., 2022).

Recently clinical trials were initiated targeting proteins encoded by the two most significant AMD GWAS loci: the *ARMS2/HTRA1* locus and the *CFH* locus. Genentech is pursuing a clinical trial for inhibition of the serine protease high-temperature requirement protein A1 (HtrA1). A Phase 1 study demonstrated safety, tolerability, pharmacokinetics (PK), and pharmacodynamics (PD) of intravitreal (ITV) aHtrA1 following single and multiple doses in patients with GA secondary to AMD (Khanani et al., 2021) (Supplementary Fig. 4). A Phase 2 study evaluating the efficacy of ITV aHtrA1 is currently in progress. Gemini Therapeutics is pursuing a clinical trial for supplementation of recombinant full length human FH protein (GEM103) locally in the eye by ITV. A phase I clinical study (NCT04246866) in advanced GA patients established safety and tolerability of GEM103. A Phase IIa study (ReGAtta) with monthly GEM103 IVT administration in GA patients confirmed safety and tolerability (Maturi et al., 2021), and demonstrated extended regulation of pathogenic AP activity (as measured by reductions in C3a and Ba protein levels) in aqueous humor over the treatment duration (Supplementary Fig. 5).

## 9. Genetic testing and polygenic risk scores for AMD

Current knowledge of AMD-associated genetic variants can be used to design genetic tests that predict the risk for developing AMD and for disease progression. Considering that many common and rare genetic variants at a large number of loci have been associated with AMD, only a comprehensive genotype assay including all risk variants will identify the total genetic risk accurately. Genetic testing for AMD is a contentious area, and the currently available tests are limited to a low number of genetic variants and vary in their predictive ability (Buitendijk et al., 2014). Recently, the EYERISK consortium developed a genetic test for AMD that allows genotyping of all 52 AMD-associated genetic variants, rare variants in AMD-associated genes including several complement genes (*CFH*, *CFI*, *C3*, *C9*), and genes that are involved in the pathogenesis of inherited macular dystrophies because the phenotype of some of these dystrophies can mimic AMD, such as *ABCA4* (a cause of Stargardt disease) and *PRPH2* (a cause of central areolar choroidal dystrophy) (de Breuk et al., 2021a).

Weighted polygenic risk scores (PRS) were calculated to assess the collective risk of the 52 independently associated variants from the IAMDGC GWAS (Fritsche et al., 2016). A significantly higher PRS was observed in individuals with late AMD compared to patients with early or intermediate AMD and control individuals (Supplementary Fig. 6), but one cannot completely distinguish the three groups based on PRS alone. The genetic test also identified a higher occurrence of rare loss-of-function variants in the *CFH*, *CFI*, and *C3* genes in late AMD patients compared with control individuals. Furthermore, the study highlighted the importance of sequencing of *PRPH2* and *ABCA4* genes since in several patients both genotype and a reevaluation of the phenotype pointed towards an inherited macular dystrophy, rather than AMD. Genetic testing can thus identify individuals who have a low, intermediate or high genetic risk for AMD, but it will not be able to differentiate AMD patients from non-AMD individuals, limiting the applicability of PRS as a screening tool. However, genetic testing can identify carriers of variants in complement genes that could be amendable for new (targeted) treatments that are currently in development for AMD (e.g. *CFI* gene therapy and FH supplementation), and genetic testing can avoid misdiagnosis of inherited macular dystrophies (de Breuk et al., 2021a).

PRS can also be used to quantify genetic risk among different disease pathways. In a recent study, pathway-specific PRS were calculated for the complement system (based on risk variants in the *CFH*, *CFI, C9, C2, TMEM9*7/VTN, and *C3* genes), the lipid metabolism (based on risk variants in *ABCA1, LIPC, CETP*, and *APOE*), and the extracellular matrix (based on risk variants in *COL4A3, ADAMTS9-AS2, COL8A1, VEGFA*, and *SYN3/TIMP3*) (Colijn et al., 2021). The complement pathway PRS was positive (>0) in 55% of participants of population-based studies, which indicates that more genetic risk than protection is present in this pathway. Most (85%) participants showed a positive PRS in 2 or more pathways, indicating that in the majority of patients genetic risk in multiple pathways underlie AMD risk. This signifies the complex cause of AMD, and suggests that in the majority of AMD patients multiple pathways may need to be targeted to achieve treatment success.

Next to a PRS based on genetic variants, also a lifestyle score was calculated based on AMD-associated lifestyle factors: smoking, vegetable intake, fruit intake and fish intake (Colijn et al., 2021). For each PRS category (low, intermediate or high), an unfavorable lifestyle increased the risk for AMD. Lifestyle increased the risk 2 to 2.3 times depending on the genetic risk. In the highest genetic risk group, the odds ratio increased from 14.9 to 35 in individuals with an unfavorable lifestyle. Therefore, all persons benefitted from a healthy lifestyle, but those with a high PRS showed the strongest risk reduction. This highlights the possibilities of counteracting predicted disease outcomes with lifestyle choices, and that such interventions can be beneficial regardless of the underlying PRS that a person has. A healthy diet consisting of sufficient fruits, vegetables, and fatty fish is consumed by only a minority of the elderly (de Koning-Backus et al., 2019), and smoking is still twice as high among those with late AMD. These data highlight the need for more rigorous measures for prevention; training of doctors in behavioral change techniques and applying genetic testing for AMD risk assessment to motivate high risk individuals may be part of this (Colijn et al., 2021).

Finally, PRS can aid in predicting disease progression. A recent study developed a prediction model for incidence of advanced AMD based on PRS, phenotypic characteristics (e.g. intermediate drusen, hyperpigmentation, AREDS classification score), and lifestyle factors (e.g. smoking, pulse pressure, Mediterranean diet) (Ajana et al., 2021). This model reached a high discrimination performance with an AUC of 92% at 5 years. High-risk participants who were classified as free of AMD by AREDS classification were characterized by a high frequency of intermediate drusen at baseline, a very high PRS, and a high cumulative incidence of advanced AMD (9 incident cases of 75; 12%). Therefore, a very high PRS may help to identify patients at high risk for progression to advanced AMD. Another recent study explored the added value of genetics using a PRS in addition to the clinical severity grading at baseline as quantified by drusen detection software, to predict disease progression in AMD patients after 6.5 years of follow-up (Heesterbeek et al., 2019). The PRS was strongly associated with the drusen coverage at baseline, and both the PRS and drusen coverage were associated with disease progression. When the PRS was added as predictor in addition to the drusen coverage, R^2^ increased from 0.46 to 0.56. This improvement by the PRS was predominantly seen in patients with a drusen coverage <15%. In patients with a larger drusen coverage, the PRS had less added value to predict progression. Thus, in addition to phenotypic characteristics, PRS may aid in predicting AMD progression, where those with the highest genetic risks are at most risk to progress to late AMD. Clinical trials for arresting disease progression may become more effective by selecting patients at high risk for progression, based both on phenotypic characteristics and a high PRS.

## 10. Discussion and future directions

Currently, genetic variants located at 34 distinct loci have been shown to be associated with AMD. Variants identified in protein-coding regions established the immediate role of the complement pathway in AMD pathogenesis. However, the majority of AMD-associated genetic variants are present in non-coding regions of the genome. A limitation of current GWAS studies is that they do not evaluate mitochondrial DNA, while a mitochondrial contribution in AMD is increasingly recognized. Additional population-based strategies that identify common genetic variants with small effects, rare, highly penetrant variants, and those variants from diverse populations can advance our understanding of the functional consequences of genes with AMD initiation and progression as well as identify pathways that may be useful targets for therapies. High-throughput functional approaches that use clinical data for Mendelian randomization of biomarkers and AMD, as well as tissue- and cellular-based ex vivo platforms (including RPE and retinal organoids derived from iPS cells) are key to making the transition from association to function. Generation of large-scale data sets using omics and subsequent QTL studies can aid in bridging the gap between the association signal and its underlying disease-related molecular process. Multi-omics in large cohorts that integrate genomics, proteomics, metabolomics, lipidomics and other types of omics are needed to gain an integrated molecular understanding of the disease processes.

Single cell sequencing studies of the neural retina, RPE and choroid identified the cell types that express AMD-associated genes and are involved in the pathogenesis of AMD, in particular non-neuronal cells such as RPE, glia, myeloid and choroidal cells. Such studies have made it clear that we must consider the genetic risk factors for AMD within the contexts of their impact on specific cell populations, while keeping in mind that expression changes in one cell type may alter other cell types in mediating risk or protection from AMD. Expression QTL (eQTL) is a powerful tool for prioritizing and discovering potential AMD-causal genes, and has already pinpointed potential causal genes for several AMD GWAS loci. Studies on the effect of genetic variants in the complement system have benefitted from systemic expression of complement proteins, linking genotype with protein expression and function. Common variants at the *CFH* locus were strongly associated with systemic concentrations of FHR-1, FHR-2, FHR-3, FHR-4 and FHR-5, and Mendelian randomization analyses supported a causal role of these proteins in AMD. Extending pQTL studies to proteins outside of the complement system may aid in further understanding the effect of genetic variants and pinpointing causal genes in AMD. In addition, iPSC-RPE can be used as a model system to prioritize and functionally characterize causal variants at AMD risk loci that are acting on a local level and which may involve cell-cell interactions but are independent of the host immune system. For example, omics techniques including RNA-seq, ATAC-seq and ChIP-seq have provided insight into the effect of genetic variation at the *VEGFA* AMD GWAS locus. Extending these studies to iPSC-RPEs and other AMD-relevant cell types from hundreds of different individuals could lead to the identification and functional characterization of the causal variants in many more of the AMD risk loci.

Several clinical trials targeting proteins encoded by AMD GWAS loci (*C3*, *CFB*, *CFI*, *CFH*, *ARMS2/HTRA1*) have been completed or are currently ongoing. Some success was seen in a recent phase III clinical trial for C3 inhibition (APL-2, pegcetacoplan), which showed a modest reduction in GA lesion growth. Recently clinical trials were initiated targeting proteins at the two most significant AMD GWAS loci: the *ARMS2/HTRA1* locus and the *CFH* locus. A Phase 1 study by Genentech investigated the safety, tolerability, PK and PD of an antibody directed against HtrA1 in patients with GA secondary to AMD, and a Phase 2 study evaluating the efficacy of aHtrA1 is currently in progress. A recent eQTL study in RPE/choroid identified increased *HTRA1* mRNA levels with the AMD-risk conferring SNP at the *ARMS2/HTRA1* locus (Orozco et al., 2020), but contradictory results were observed in a recent study which showed reduced expression of *HTRA1* mRNA and protein in human donor eyes homozygous for the *ARMS2/HTRA1* risk allele (Williams et al., 2021). The latter finding suggests that *HTRA1* may need to be augmented rather than inhibited; the clinical trial data will show whether HTRA1 inhibition is the appropriate therapeutic approach. Gemini Therapeutics completed phase I and phase II clinical studies for supplementation of recombinant FH protein (GEM103) locally in the eye, which confirmed safety and tolerability, and demonstrated extended regulation of pathogenic complement activity in aqueous humor. A sham-controlled efficacy study for FH supplementation is now planned. A unique aspect of the GEM103 (FH supplementation, Gemini Therapeutics) and GT005 (*CFI* gene therapy, Gyroscope Therapeutics) clinical trials is that patients are selected for carrying risk variants in *CFH* and *CFI*, respectively, before inclusion in the trials. Patient selection based on genotype has not been performed in any other of the clinical trials for AMD so far, and results of these trials will show whether patient selection based on genotype leads to a higher efficacy. Future therapeutic strategies may consider the strong protective effects at the *CFH* locus, which are associated with reduced FHR protein concentrations, and have been suggested to alleviate risk at the *CFH* and *ARMS2/HTRA1* loci (Pappas et al., 2021). The failure of several clinical trials targeting components of the complement system highlights the need to better understand the local and systemic effects of the target pathway (de Jong et al., 2021), as well as the importance of selecting a therapeutic endpoint that is reflective of that pathway. A recent GWAS showed non-AMD-related genetic variants as associated with the progression of GA (Grassmann et al., 2019), which is a cautionary note that the progression of such atrophy may not be solely determined by the factors that are associated with AMD.

In terms of genetic risk profiling for AMD, considerable progress has been made at predicting AMD risk and progression in the general population. Such efforts can already have immediate benefits in the design and implementation of clinical trials to evaluate efficacy in delaying or preventing disease incidence and/or progression. Selection of high-risk cohorts can greatly reduce sample sizes and the duration of clinical trials to show a benefit and yet the findings may well be generalizable to the entire AMD cohort just as we have seen from the demonstration that all at-risk individuals over the entire PRS range of values can benefit from dietary interventions. Whether or not to recommend individual AMD genetic testing based on the PRS score as developed by EYERISK and which will continue to evolve as more rare, highly penetrant AMD-related variants are identified in diverse populations, remains controversial. At this time, we lack a variety of treatments that can be selected for efficacy based on the genetic risk determinants. Clinical management of AMD (particularly exudative AMD) is largely driven by the patient’s clinical responses to the currently available therapies, but this could change as new treatment options emerge.

There is little doubt that the large GWAS studies of AMD represent a turning point in our exploration of this condition. The results of those studies, while now serving as a driving force for several clinical trials, also engage to expand our concepts of systems genomics to tackle the many questions that have been raised by the genetic loci and pathways that have been implicated from these studies. We have a wide range of new tools and approaches for deciphering function and causality from the associations that have been reported. We are increasingly appreciative of the intertwining of genetics with the interactions of the multiple cell types and gene expression patterns that are critical for human vision. There remains a great deal to be done and there is a great deal of promise for developing therapies that will lessen the burden of AMD.

## Supplementary Material

Supplementary Figure 5

Supplementary Figure 6

Supplementary Figure 4B

Supplementary Figure 4A

Supplementary Figure 3

Supplementary Figure 2

Supplementary Figure 1

## Data Availability

No data was used for the research described in the article.
