# Supplementary figures and images for: Systems genomics in age-related macular degeneration
Source: Exp Eye Res. Author manuscript; Available in PMC 2023 May 1. (PMC10150562; doi:10.1016/j.exer.2022.109248)

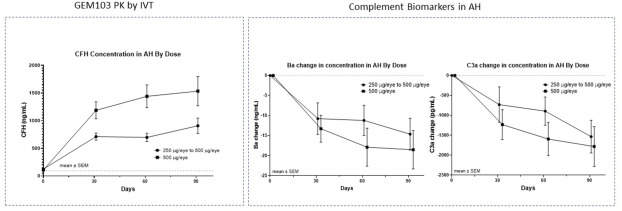

Supplement: Supplementary Figure 5 [file NIHMS1879849-supplement-Supplementary_Figure_5.jpg]

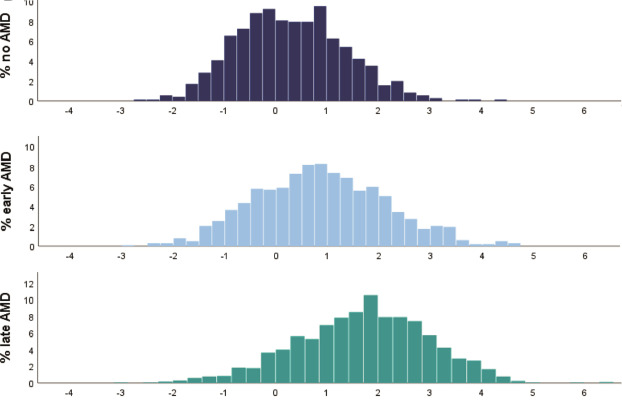

Supplement: Supplementary Figure 6 [file NIHMS1879849-supplement-Supplementary_Figure_6.jpg]

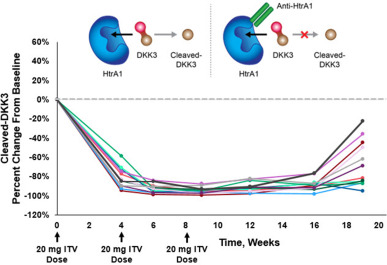

Supplement: Supplementary Figure 4B [file NIHMS1879849-supplement-Supplementary_Figure_4B.jpg]

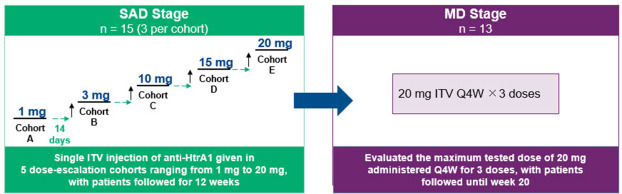

Supplement: Supplementary Figure 4A [file NIHMS1879849-supplement-Supplementary_Figure_4A.jpg]

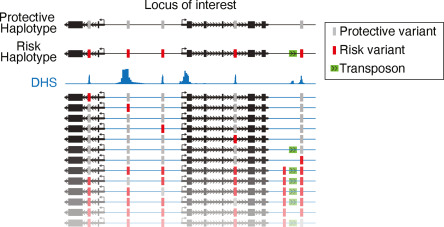

Supplement: Supplementary Figure 3 [file NIHMS1879849-supplement-Supplementary_Figure_3.jpg]

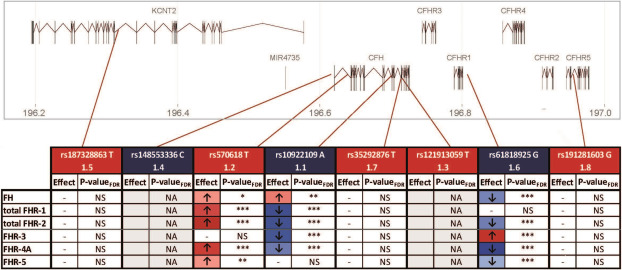

Supplement: Supplementary Figure 2 [file NIHMS1879849-supplement-Supplementary_Figure_2.jpg]

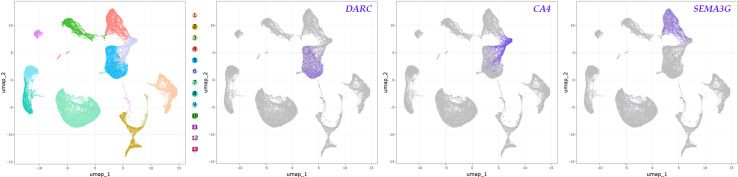

Supplement: Supplementary Figure 1 [file NIHMS1879849-supplement-Supplementary_Figure_1.jpg]
